# Expression of concern: HDACI regulates the PI3K/Akt signaling pathway to reverse MCF-7/PTX resistance by inhibiting SET

**DOI:** 10.1039/d4ra90094d

**Published:** 2024-09-04

**Authors:** Weipeng Zhang, Xiaowei Zheng, Ti Meng, Haisheng You, Yalin Dong, Jianfeng Xing, Siying Chen

**Affiliations:** a Department of Pharmacy, The First Affiliated Hospital of Xi'an Jiaotong University No. 277 of Yanta West Road Xi'an Shaanxi 710061 PR China ychen0326@163.com +86-29-85323240 +86-29-85323243; b Department of Pharmacy, The Eighth Hospital of Xi'an Xi'an Shaanxi 710061 PR China; c School of Pharmacy, Xi'an Jiaotong University No. 76 of Yanta West Road Xi'an Shaanxi 710061 PR China xajdxjf@mail.xjtu.edu.cn +86-29-82655130 +86-29-82655130

## Abstract

Expression of concern for ‘HDACI regulates the PI3K/Akt signaling pathway to reverse MCF-7/PTX resistance by inhibiting SET’ by Weipeng Zhang *et al.*, *RSC Adv.*, 2016, **6**, 48072–48082, https://doi.org/10.1039/C6RA06423J

*RSC Advances* is publishing this expression of concern in order to alert readers that concerns have been raised regarding the western blot images in Fig. 2a and b, 4a and b, 5c, and 6a and b. The authors have been asked to provide the raw data for these concerns but have not been able to do this. An expression of concern will continue to be associated with the article until we receive conclusive evidence regarding the reliability of the reported data.

Laura Fisher

23rd August 2024

Executive Editor, *RSC Advances*

